# Carpe Diem: Genetic Testing for Familial Hypercholesterolemia From Acute Coronary Care to the Community

**DOI:** 10.1016/j.jacadv.2026.103100

**Published:** 2026-08-15

**Authors:** Jing Pang, Robert S. Rosenson, Michael J. Wilkinson, Dinesh K. Kalra, Gerald F. Watts

**Affiliations:** a Medical School, University of Western Australia, Western Australia, Australia; b Metabolism and Lipids Program, Mount Sinai Fuster Heart Hospital, Icahn School of Medicine at Mount Sinai, New York, New York, USA; c Division of Cardiovascular Medicine, Department of Medicine, University of California San Diego, La Jolla, California, USA; d Division of Cardiovascular Medicine, Department of Medicine, University of Louisville, Louisville, Kentucky, USA; e Department of Cardiology, Royal Perth Hospital, Western Australia, Australia

**Keywords:** acute coronary syndrome, familial hypercholesterolemia, genetic testing

Familial hypercholesterolemia (FH), an autosomal semidominant condition, is among the most common inherited causes of premature atherosclerotic cardiovascular disease (ASCVD), affecting ∼1 in 300 individuals worldwide,^1^ but over 90% remain undetected and uncared for. Untreated FH confers a lifetime of markedly elevated low-density lipoprotein cholesterol (LDL-C) exposure, translating into accelerated ASCVD.^2^ For many individuals, an acute coronary syndrome (ACS) at an early age is the first clinical signal in FH. International guidelines recommend the integration of selective, opportunistic, and universal methods for early detection, with downstream diagnostic, risk stratification, and treatment pathways.^2^ Identification of probands with ACS and subsequent cascade testing of close relatives, with the assessment of FH causative gene variants and LDL-C concentrations coupled with genetic counseling, is strongly advised. The urgency for early diagnosis and prompt LDL-C lowering with combination therapy is far greater in homozygous than heterozygous FH.^2^

In this issue of *JACC: Advances*, Vaizman et al^3^ report the results the ACCURATE (Advancing Cardiac Care Unit-based Rapid Assessment and Treatment of HypErcholesterolemia) study from Vancouver, Canada. This was a nonrandomized, prospective 2-phase cohort study designed to evaluate whether opportunistic genetic testing at the time of an ACS increases FH diagnosis rates and optimizes downstream cholesterol-lowering therapy. Enrolling patients <60 years of age admitted with ACS and an LDL-C ≥4 mmol/L (≥155 mg/dL), the investigators compared outcomes between a standard-of-care cohort (phase 1, n = 40) and a genetic testing cohort in which results were returned to treating physicians (phase 2, n = 100). The primary endpoint of a new diagnosis of definite FH was reported in 10% of phase 2 patients vs none in phase 1. Beyond diagnosis, the clinical benefits extended to treatment: patients in phase 2 were significantly more likely to be receiving statin-ezetimibe combination therapy (52% vs 30%), to be on statin therapy (95% vs 83%), and to have attained an LDL-C <1.8 mmol/L (90 mg/dL) (73% vs 53%) at 15 months. These findings position the embedding of opportunistic genetic testing in coronary care units and beyond as a potential driver of effective and sustained therapy for patients newly diagnosed with FH.

Although not the first study to have genetically tested patients for FH with ACS in an acute cardiac care setting, the ACCURATE study makes several important contributions. Opportunistic testing at the time of premature ACS (<55 years of age in men, <60 years of age in women) has the potential to identify previously unrecognized FH probands, the anticipated frequency being ∼1 in 15 in this setting. Importantly, such testing should also lead to cascade testing of close family members in the community. The pragmatic, real-world application of embedding genetic testing within a standard clinical pathway and its effectiveness suggests the approach may be generalizable. The return of results directly to treating physicians, rather than via the pathway of clinical genetics services, is a practical and potentially scalable model that needs further implementation research. Critically, the study demonstrates that the diagnostic yield of genetic testing translates prospectively into measurable clinical benefit, extending previous FH prevalence studies in ACS.

The ACCURATE study has limitations, several of which the authors acknowledge and discuss. The nonrandomized sequential cohort design introduces the possibility of temporal confounding and the relatively modest sample size, particularly in phase 1 of the study, limits the statistical precision of between-group comparisons. The study population was predominantly of European ancestry, and it remains uncertain whether diagnostic yield and treatment response would differ across more diverse populations, where knowledge of FH-causing variants and variant databases are less complete. More importantly, the study does not report cascade testing rates among first-degree relatives of the identified probands; this is arguably the most cost-effective downstream outcome of any FH detection strategy that would support its implementation.^2^ The full public health benefit of opportunistic detection of probands cannot be realized without systematic testing of close family members, with particular emphasis on children, adolescents, and young adults. Future studies of an evolving model of care must systematically assess implementation, service, and patient outcomes. Among these, patient-reported experience and outcomes measures are paramount. Adoption, penetrance, and scalability for implementation and timeliness, equity and cost-effectiveness for service delivery are key considerations.

The findings of the ACCURATE study have practical implications that extend well beyond the acute cardiac care setting. The identification of an FH proband at the time of premature ACS initiates a chain of clinical responsibility: the interventional cardiologist or acute physician must flag the diagnosis; a lipidologist or clinical geneticist must confirm variant pathogenicity and coordinate cascade testing; and primary care must absorb the ongoing clinical management and cascade testing, depending on the context of the national, regional, or local model of care for FH adopted by a given health service. Integrated multidisciplinary pathways for FH may not exist in many health systems. The model of care in which genetic test results are returned to the treating physician is appealingly simple, but the translation of a genetic result into cascade testing requires skills and capabilities that should preferably involve genetic counselors or lipidologists with expertise in genetics.^2^^,^^4^^,^^5^ A clear, funded pathway for postdischarge genetic counseling and family notification is an essential prerequisite of good clinical practice. Health systems that have invested in FH registries and cascade testing infrastructure are best positioned to capitalize on opportunistic testing strategies;^2^ where that infrastructure is absent, the risk is that the genetic diagnosis becomes an endpoint, rather than the starting point for broader family benefit that entails effective reduction of LDL-C with proven therapies and mitigation of other cardiovascular and adverse behavioral risk factors.

There are other important considerations beyond genetic testing for FH.^6^ Although the presence of a gene variant signifies increased ASCVD risk, at least 1 in 5 patients with a frank phenotypic diagnosis of FH are not at present reported to bear a pathogenic or likely pathogenic variant, mainly related to the analytical limitations of current testing panels.^3^^,^^7^ This means that equity of care mandates that patients with a frank clinical diagnosis of FH in the absence of a positive genetic result should be advised accordingly and cholesterol testing offered to close family members, with appropriate family counseling.^2^ The other matter, which was not reported in the present study, concerns elevated plasma lipoprotein(a) (Lp[a]). Briefly, current guidelines recommend that all patients with FH, and indeed all adults in the population, be tested for Lp(a) and consideration also given to cascade testing close relatives of probands with elevated Lp(a).^2^^,^^8^^,^^9^ Such approaches may be more persuasive and adoptable with the outcome of large cardiovascular outcomes trials of therapies targeted at Lp(a) lowering.

In aggregate, the ACCURATE study offers a compelling proof-of-concept: opportunistic genetic testing at the time of premature ACS is feasible, diagnostically productive, and associated with enhanced use of cholesterol-lowering therapy and attainment of LDL-C goals. An admission with a premature ACS represents a high-yield clinical setting that the cardiology community has been slow to leverage for the application of genomic medicine to coronary prevention. Implementing FH genetic testing within a standard acute care pathway is a potentially credible and highly valuable model for practice.^2^^,^^3^ The challenge is to design the clinical and health system infrastructure that converts a genetic diagnosis into a cascade of preventive benefit for the families of those who survive their first coronary event. That said, the implementation of the model will require strategies that should effectuate changes in the perspectives and behaviors among patients, health care professionals, organizations, and policy makers concerning the importance of genomic medicine.^2^ Sustained financing of the model is critical, but so is increasing public awareness of the importance of FH and genetics, as well as enhancing the requisite capabilities of health care professionals across the spectrum of care of FH, starting in the present context in the acute cardiac unit and extending into primary care and the community. Hence, the ACCURATE study is a small but innovative pilot study with very broad implications, with much model design and implementation research to be undertaken on the road ahead.^2^

## Funding support and author disclosures

Dr Rosenson has received research funding to his institution from 89Bio, Amgen, Argo Biopharma, Arrowhead, AstraZeneca, Kalexo Bio, Lilly, Mammoth Biosciences, Merck Sharp and Dohme, NIH, Novartis, Novo Nordisk, and Verve Therapeutics; consulting fees from General Medicines, GSK, Lipigon, Novo Nordisk, Amgen, Novartis, and Rona Therapeutics; royalties from Wolters Kluwer (UptoDate); and stock holdings from MediMergent, LLC. Dr Wilkinson is a consultant to Amarin, Regeneron, Kaneka, Ionis, and NewAmsterdam; has received advisory board fees from Novartis, Regeneron, Ionis, Arrowhead, Lilly, and NewAmsterdam; has received grant support from Amgen and Esperion (investigator-initiated studies); has received support through an institutional consulting agreement with Novartis paid to his institution for advising on research; and has contracted research grants to his institution with Amgen, Novartis, Ionis, Lilly, Silence, and Mineralys. Dr Watts has received research grants, consultancy fees, and lecture honoraria from Amgen, Arrowhead Pharmaceuticals, and Novartis. All other authors have reported that they have no relationships relevant to the contents of this paper to disclose.
